# Bioprinting of skin constructs for wound healing

**DOI:** 10.1186/s41038-017-0104-x

**Published:** 2018-01-23

**Authors:** Peng He, Junning Zhao, Jiumeng Zhang, Bo Li, Zhiyuan Gou, Maling Gou, Xiaolu Li

**Affiliations:** 1 The Affiliated Hospital of Southwest Medical University, the department of Plastic & Burns Surgery, Tai Ping Street, Luzhou, 646000 People’s Republic of China; 2 State Key Laboratory of Biotherapy and Cancer Center, West China Hospital, Sichuan University, Chengdu, 610041 People’s Republic of China; 3 Collaborative Innovation Center for Biotherapy, Chengdu, 610041 People’s Republic of China; 4 Sichuan Academy of Chinese Medical Sciences, Sichuan Translational Medicine Center of Chinese Medicine, Ren Min Nan Lu Road, Chengdu, 610041 People’s Republic of China

**Keywords:** Bioprinting, Skin constructs, Wound healing, Bioink

## Abstract

Extensive burns and full-thickness skin wounds are difficult to repair. Autologous split-thickness skin graft (ASSG) is still used as the gold standard in the clinic. However, the shortage of donor skin tissues is a serious problem. A potential solution to this problem is to fabricate skin constructs using biomaterial scaffolds with or without cells. Bioprinting is being applied to address the need for skin tissues suitable for transplantation, and can lead to the development of skin equivalents for wound healing therapy. Here, we summarize strategies of bioprinting and review current advances of bioprinting of skin constructs. There will be challenges on the way of 3D bioprinting for skin regeneration, but we still believe bioprinting will be potential skills for wounds healing in the foreseeable future.

## Background

Annually, patients with extensive burns and full-thickness skin wounds suffer substantial burdens, including physical, psychological, economical, individual and social difficulties [1] (as is showed in the Table 1 [2]). Therefore, it is necessary to highlight innovant techniques in crossing fields. Severe clinical practice in the treatment of burn injury has been developed to the application of tissue engineering skin substitutes for the stage, these tissue engineered skin substitutes are often used to assist the wound closure and/or by improving the function and cosmetic effect in order to achieve the purpose of improving the quality of life of long-term. However, the current tissue engineering technology is not able to produce a truly functional skin substitute at a reasonable cost [3–6]. Although advances have been made recently in treating these wounds, autologous split-thickness skin graft (ASSG) remains the gold standard in the clinic for large wounds. ASSG involves removing a piece of skin from a secondary surgical site for the patient, stretching the skin, and reapplying the graft on the impaired skin. However, ASSG is limited by the number and size of donor sites [7]. Skin bioprinting may provide a novel alternative to ASSG therapy. The availability of skin constructs fabricated by bioprinting using in vitro expanded cells from skin biopsy would alleviate the problem of shortage of donor sites in ASSG. The process of skin bioprinting involves collecting skin tissues from patients by skin biopsy and culturing them in vitro to obtain enough number of cells; Cultured skin cells are then mixed with biomaterials and delivered to a three dimensional (3D) bioprinter for fabrication of customized skin [8].

**Table 1:** Healthcare costs of burn patients in high-income countries (converted to US dollars, 2012) [2]

	Mean($)	Range($)	Median($)
Costs per burn center day	2705	111–11,607	2060
Costs per burn center ICU day	3164	1590–4657	2969
Costs per general hospital day	1959	585–4314	1468
Costs per general ICU day	4356	4356	4356
Total healthcare costs/p t	88,218	704–717,306	44,024
Flame	87,140	50,508–109,469	94,291
Scald	33,960	15,882–32,526	33,981
Electric	55,281	26,076–70,311	69,457
Costs per 1% TBSA	4159	162–20,663	2633

*ICU* intensive care unit, *p t* patient, *TBSA* total body surface area

## Review

### Bioprinting technology and wound healing

Bioprinting is an additive manufacturing technology, which can deposit living cells, biomaterials and factors in the complex 3D constructs [7]. It provides a high degree of flexibility and repeatability using a computer-controlled 3D printer to fabricate 3D structures via a layer-by-layer printing process. Bioprinting generally contains the following three steps [8–12]*.* Firstly, collecting accurate information of tissues and organs for the model designation and materials selection; secondly, transferring the information into electrical signal to control the printer to print the tissues; thirdly, creating a stable structure.

**Fig. 1:**
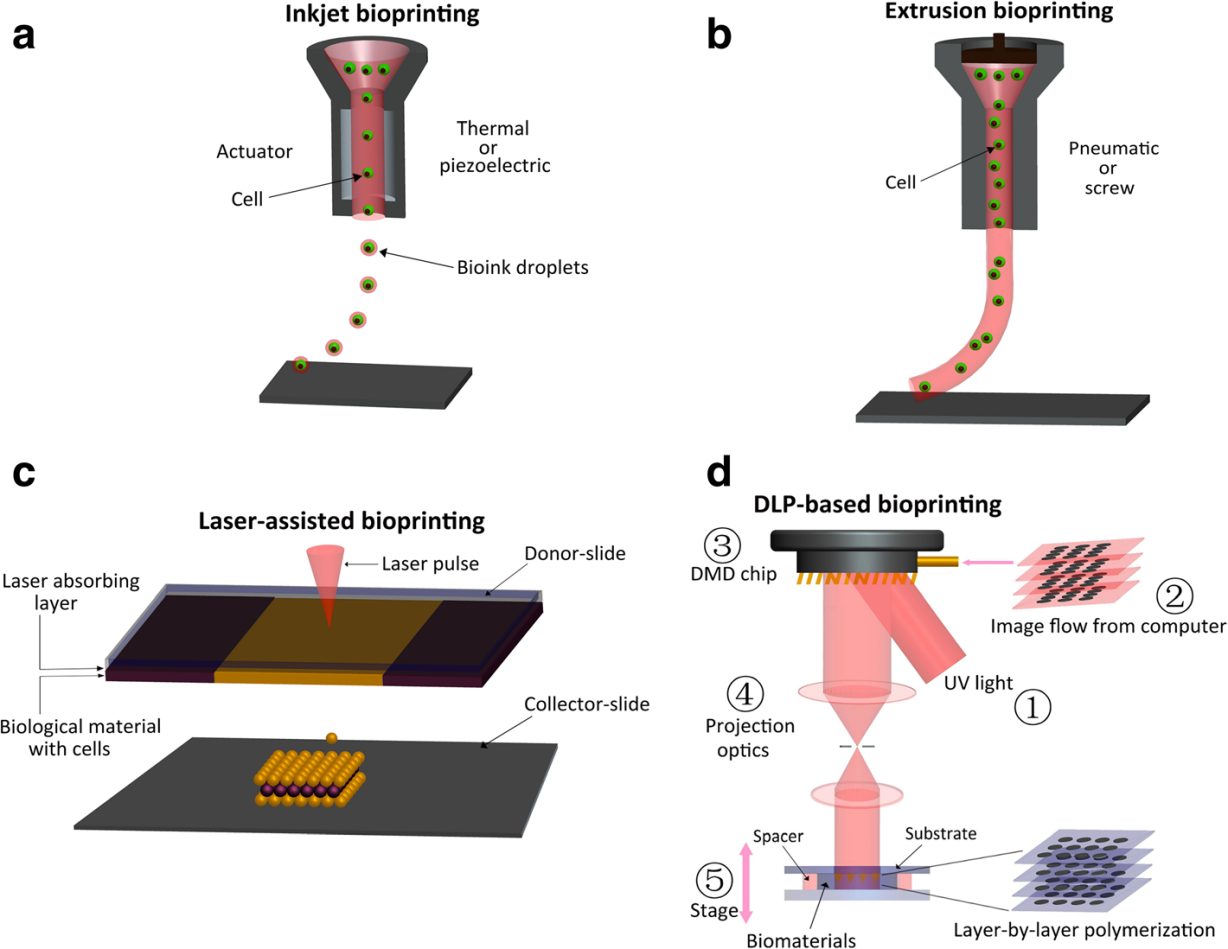
Bioprinting techniques. **a** Inkjet bioprinter eject small droplets of cells and hydrogel sequentially to build up tissues. **b** Extrusion bioprinter use pneumatics or manual force to continuously extrude a liquid cell–hydrogel solution. **c** Sketch of the laser printer setup. **d** Schematic of the DLP based bioprinter—dynamic optical projection stereolithography (DOPsL)

There are many kinds of bioprinting technologies, four (Fig. 1) of which are widely used at present: Inkjet-based printing [13], Extrusion-based printing [13], Laser-assisted printing [14], DLP-based printing—dynamic optical projection stereolithography (DOPsL) [15], and key differences between these four printing technologies are described in Table 2 [16]. Cell viability can be affected by several factors, including bioprinting technique used, the printing speed, and the species of seeding cells [13–16].

**Table 2:** Comparison of the different bioprinting techniques discussed in this review [16]

	Inkjet printing	Extrusion printing	Laser-assisted printing	DLP printing
Printing process	Serial (drop by drop)	Serial (line by line)	Serial (dot by dot)	Parallel and continuous (projection based)
Printing speed	Medium (mm/s)	Slow (10–50 um/s)	Medium (mm/s)	Fast (mm^3^/s)
Resolution	50 um	5 um	< 500 nm	1 um
Cell viability	> 85%	40–80%	> 85%	85–95%
Material choice	Thermo/pH/photo-sensitive	Thermo/photo-sensitive	Photosensitive	Photosensitive

**Fig. 2:**
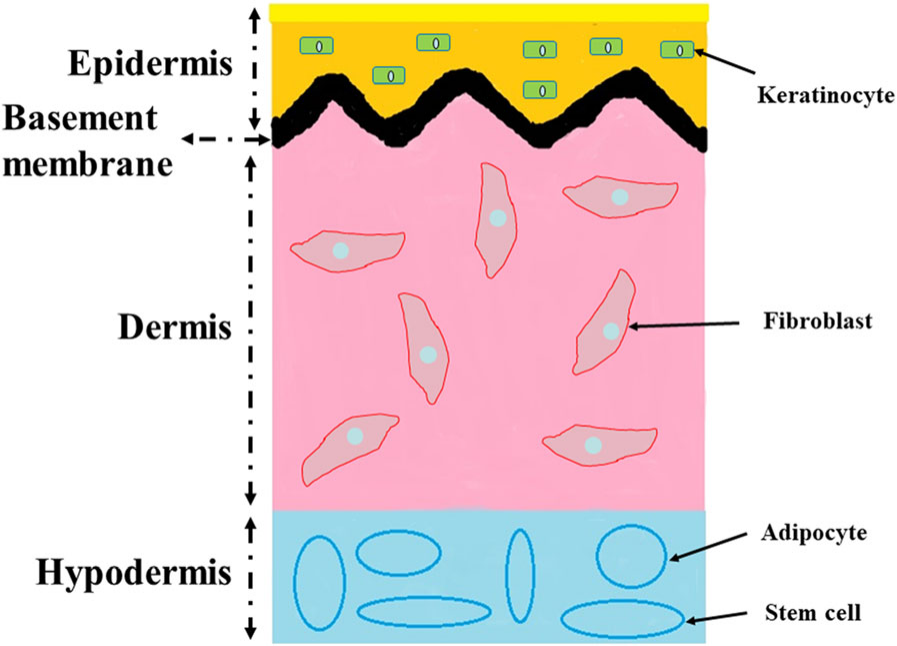
The structure of skin [28]. It consists of four layers: the epidermis, the basement membrane, the dermis, and the hypodermis

Wound healing is a complex procedure, involving several distinct stages and a series of cells and cytokines [17]. To facilitate the wound healing process, a range of natural biomaterials have been developed, namely cellulose, alginate, collagen and chitin, hyaluronic acids, and others [18–26]. Because of the favorable characteristics of natural biomaterials, such as biocompatibility, biodegradation, low-toxicity or nontoxicity, mechanical stability, high moisture content, and high availability, the use of natural biomaterials is attractive for advanced wound management. In addition, C-Periodate nanocellulose is suitable for use as “bioink” for printing 3D porous structures [27]. The availability of suitable biomaterials and advances in bioprinting technologies demonstrates that bioprinting can be successfully utilized for the fabrication of novel wound dressings. In addition, these wound dressings have the capability of maintaining a moist microenvironment and minimizing bacterial infection. However, because of no structure or function of the human native skin, these dressings cannot reconstruct the vessel networks, deliver the nutrition and oxygen, and remove wastes. In the contrary, they may generate immunological rejection for the xenogenous materials. So, it is vitally important to find a better measure to reconstruct the function and structure of the native skin. As well as being used for creating organs, bioprinting is also used to create skin equivalents for graft. Skin can be modeled as a 3D structure consisting of multiple 2D constructs: subcutaneous tissue, dermis, and epidermis (the structure of skin is showed in Fig. 2 [28]), each of which contains multiple cell types arranged in precise spatial configurations. Skin bioprinting is a natural evolution of bioprinting technology [29].

### Skin bioprinting

In vitro and in situ bioprinting are two basic styles for skin bioprinting.

#### In vitro bioprinting

Mouse NIH3T3 Swiss albino fibroblast (DSMZ Braunschweig, Germany) and human immortalized HaCaT (DKFZ, Heidelberg, Germany) keratinocyte cell lines were used to print 3D skin constructs [14, 30]. These well-established cell lines were also combined in other studies [31, 32]. Because of secreting growth factors supportive for keratinocytes, three T3 fibroblast cells are usually utilized to cultivate keratinocytes [33–35].

Collagen is the main extracellular matrix (ECM) protein in skin. Collagen type I, from rat tail, was used as hydrogel embedding the cells for the printing process and as ECM afterwards, to approximate native skin as far as possible [14, 36, 37].

**Fig. 3:**
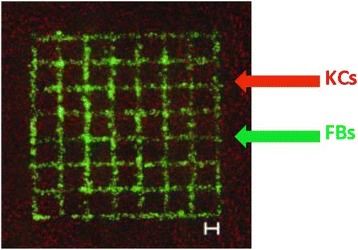
A structure of fibroblasts (green) and keratinocytes (red) was printed by the laser printing technique [14]

**Fig. 4:**
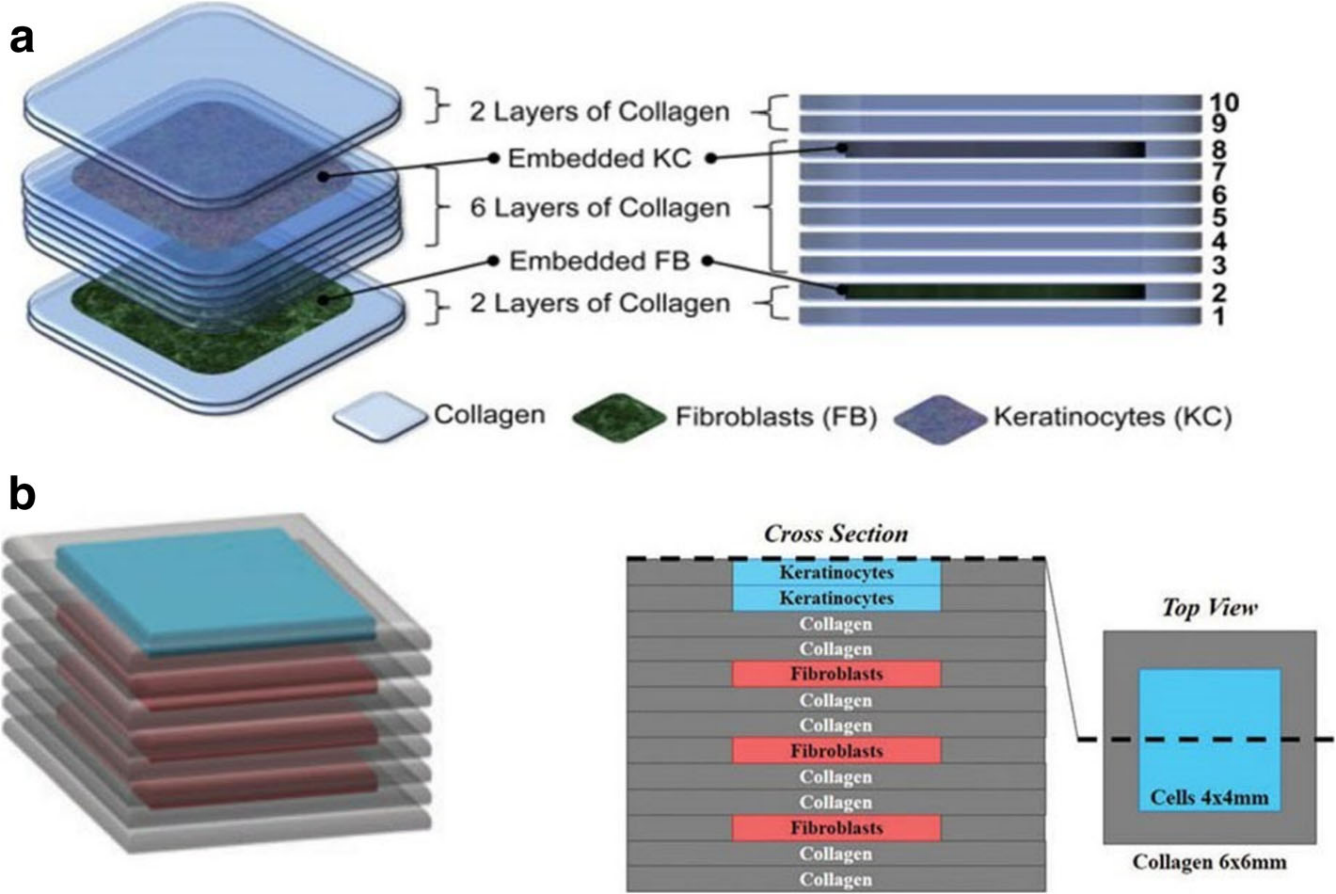
Constructs with the multi-layered skin cells and collagen were printed by an extrusion printer via layer-by-layer [36, 37]. **a** Fibroblasts were printed in the 2nd collagen layer, and six layers of collagen were printed over the fibroblasts. Keratinocytes were printed in the 8th layer of collagen and two layers of collagen were used to cover the keratinocytes layer. **b** The printed skin structure contains eight collagen layers. These include six collagen layers alternating with three layers of fibroblast layers and two collagen layers separating the stacked fibroblast layers from keratinocytes

**Fig. 5:**
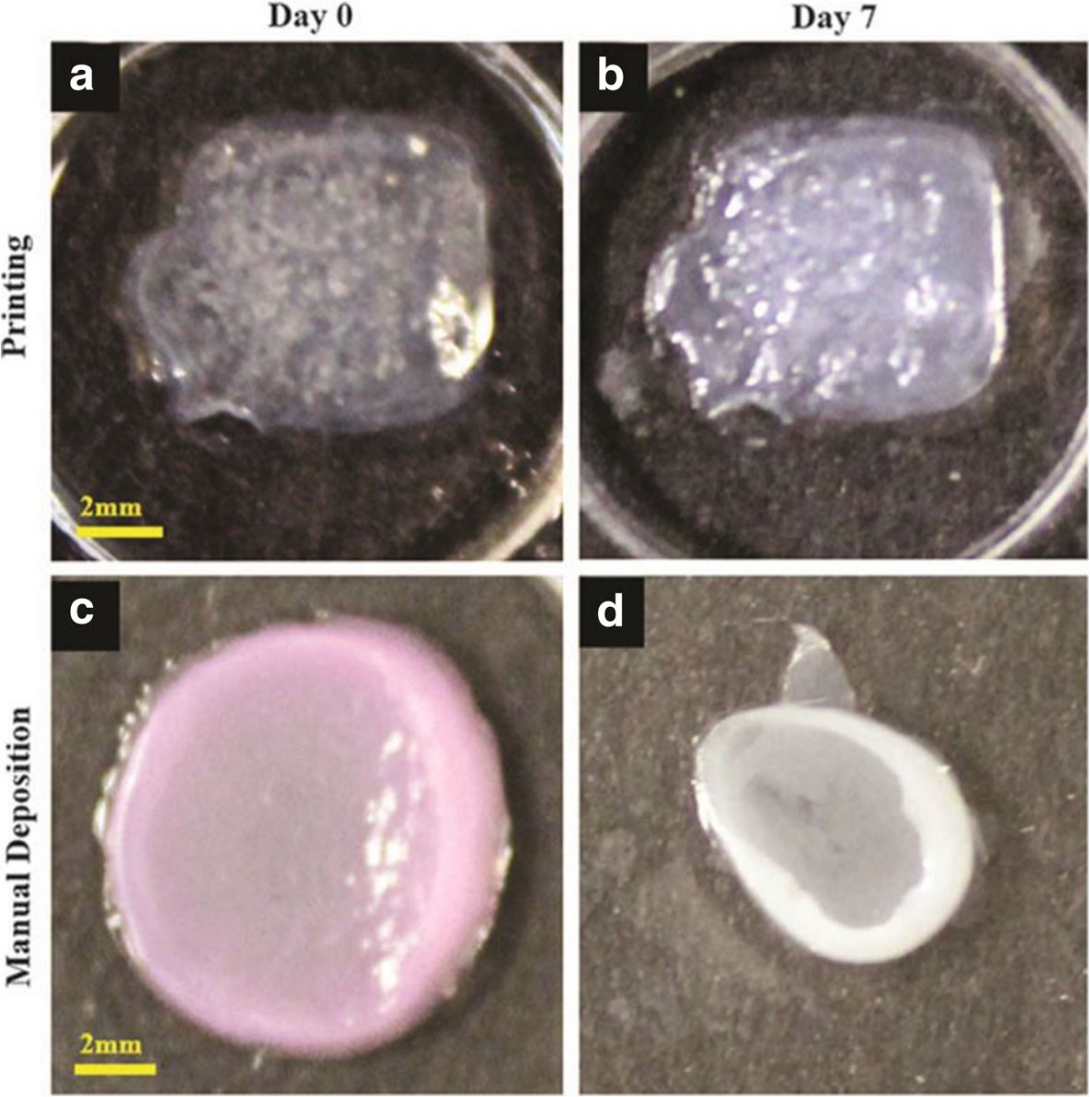
SShape and form of printed skin tissue. A comparison of skin tissues fabricated via 3D bioprinting and manual deposition under submerged culture condition after 7 days [37]. **a**, **b** 3D printed structures retain their form (dimensions) and shape. **c**, **d** Manually deposited structures shrink and form concave shapes (buckle) under submerged culture condition after 7 days

A study [14] demonstrated that 20 layers of fibroblasts (murine NIH-3 T3) and 20 layers of keratinocytes (human HaCaT) embedded in collagen were printed by a Laser-assisted BioPrinter (LaBP) on a sheet of Matriderm® (decellularized dermal matrix) (Fig. 3), to generate simple 3D skin equivalents with dermis and epidermis-like structure. The researchers labeled the fibroblasts and keratinocytes using fluorescent cell membrane markers. The result of fluorescence microscopic images of 3D printed fibroblasts and keratinocytes showed that their bi-layered construct generates a dermis and epidermis. And after the printed skin constructs were cultivated for 10 days, it showed that connexin 43 (Cx43) was still preserved in the epidermis, demonstrating formation of gap junctions [38]. In another study [36], dermal/epidermal-like distinctive layers (Fig. 4a) were successfully printed by an extrusion printer with primary adult human dermal fibroblasts and primary adult human epidermal keratinocytes in a 3D hydrogel scaffold. Ten layers of type I collagen precursor (rat tail origin, BD Biosciences, and MA) were printed. These constructs were able to generate dermis and epidermis structures. However, this printed construct did not show tissue generation or the establishment of intercellular junctions [39]. A recent study [37] demonstrated that in vitro skin substitutes (Fig. 4b) were printed by bioprinting fibroblasts ((HFF-1) and keratinocytes (HaCaT) on collagen layers as the delivery matrix. Printed skin samples (Fig. 5a, b) retained their form (dimensions) and shape, whereas manually deposited structures (Fig. 5c, d) shrank and forme d concave shapes (buckle) (Fig. 5). The 3D printed skin tissue was morphologically and biologically similar to human skin tissue.

**Fig. 6:**
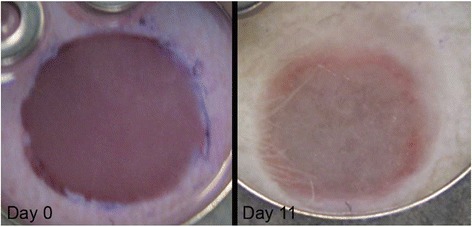
Printed skin constructs fill the full-thickness wound completely in the dorsal skin fold chamber in nude mice [30]. These constructs were fabricated via Laser-assisted BioPrinter (LaBP), including 20 layers of fibroblasts and 20 layers of keratinocytes on top of Matriderm®. The pictures show a skin construct inserted into the wound directly after the implantation (left) and on day 11 (right)

In a separate study by Michael et al. [30], similarly bi-layered constructs were fabricated in vitro, and implanted in vivo employing the dorsal skin fold chamber in nude mice (Fig. 6). These skin constructs formed dermis and epidermis. The researchers found that the printed keratinocytes formed a multi-layered epidermis with beginning differentiation and stratum corneum, and the printed fibroblasts could migrate collagen into the Matriderm® (a stabilizing matrix). Furthermore, some blood vessels from the wound bed could be observed after 11 days of transplantation.

#### In situ bioprinting

**Fig. 7:**
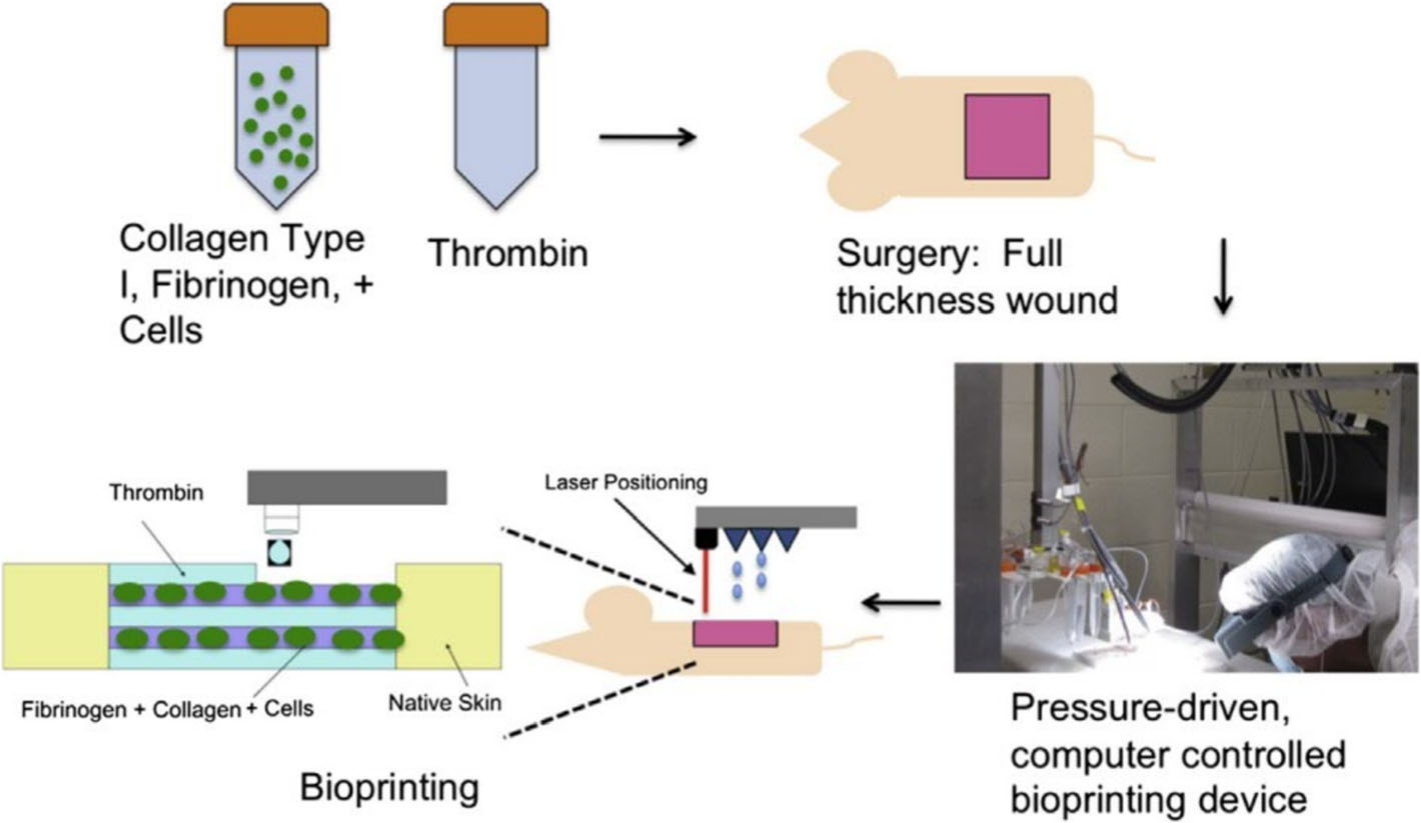
A schematic describing the approach of in situ bioprinting [41]

In the study by Binder et al.[40], the feasibility of in situ bioprinting on the dorsal defect of athymic mice using an inkjet delivery system. These researchers loaded human keratinocytes and fibroblasts into the skin printer, and printed the two cells onto a full thickness skin defect (3 cm × 2.5 cm). Fibrinogen/collagen hydrogel precursor containing fibroblasts (1.0 × 10^5^ cells/cm^2^) was the first layer and another layer of keratinocytes (1.0 × 10^7^ cells/cm^2^) above the fibroblast layer. This study demonstrated that the two different skin cells types can be directly printed onto the wound sites, and the printed constructs can mimic normal murine skin. Another study [41] directly printed amniotic fluid-derived stem cells (AFSCs) onto full-thickness skin wounds (2 cm × 2 cm) of nu/nu mice using a pressure-driven, computer controlled bioprinting device. AFSCs and bone marrow-derived mesenchymal stem cells (MSCs) were suspended in fibrin-collagen gel, mixed with the thrombin solution (a crosslinking agent), and then printed onto the wound site. The bioprinter was used to deposit two layers of a fibrin-collagen gel by depositing a layer of thrombin, a layer of fibrinogen/collagen, a second layer of thrombin, a second layer of fibrinogen/collagen, and a final layer of thrombin (Fig. 7). Even though AFSCs existed in the wound sites only for a period of time, the wound closure and re-epithelialization were increased most likely by the secretion of growth factors by MSCs.

### “Bioink” in skin bioprinting

Cells (Keratinocytes, Fibroblasts) and ECM have been combined as “bioink” for regenerating skin equivalents. They can be used to reconstruct biological structure and function of original skin tissues. Cell distribution in 3D structures can be controlled using the 3D biological printing technology to facilitate cell-cell and cell-matrix interactions. Generally, inject [42–45] and DLP [15, 46–49] bioprinting technologies are used to generate 3D cell-laden constructs [50] and complex and heterogeneous 3D tissue constructs consisting of multiple cell types [51], extrusion-based and laser-assisted printing have also been used to fabricate multilayered skin constructs.

In recent years, integration of bioprinting technologies with stem cell research has been an emerging area. Stem cells, such as human bone marrow stem cells, embryonic stem cells (ESCs) and adipose-derived stem cells (ASCs) have been reported to be work as “bioink” directly onto substrates, including the skin regeneration [52–55]**.** Due to the characteristics of stem cells have multilineage differentiation potential and self-renewal capacity, subsidiary structure can be constructed using skin epidermal stem cells such as hair follicles, sweat glands; Stem cells can also be used to regenerate skin tissue to vascular network, the establishment of cells, and cell and tissue biology. Therefore, stem cell has the potential ability to print the real structural and functional integrity of the skin substitute. Stem cell printing has a high cell survival rate, it was reported that the stem cell survival rates before and after 3D bioprinting are 97% and 94%, respectively [56–58], which effectively guarantee the possible usage of this technique for wounds healing.

### Advantages and drawbacks

**Fig. 8:**
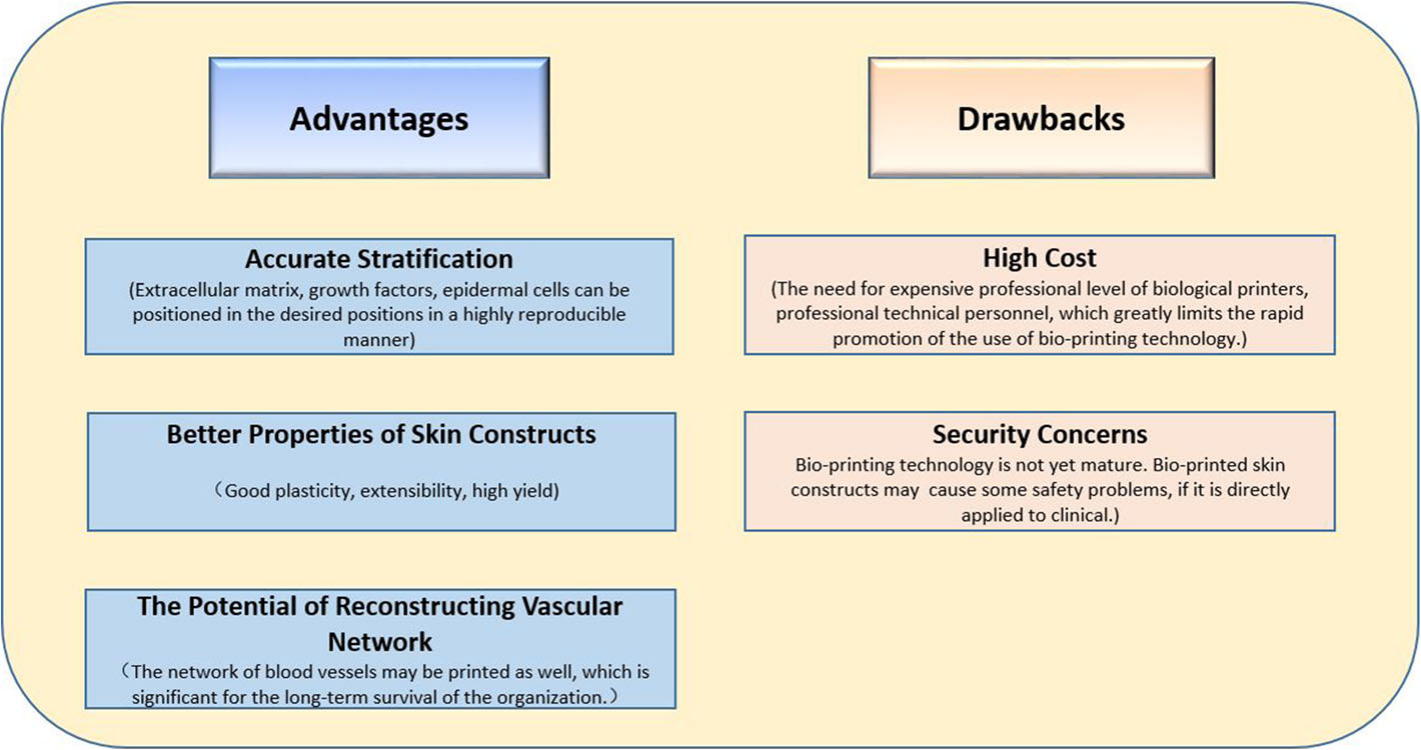
Advantages and disadvantages of skin bioprinting

Compared with tissue engineering technology, 3D printing is personalized, has advantages of flexibility, in the alternative to the accurate positioning of bioactive molecules and other advantages, such as improving the skin construction speed and shortening the patient waiting time, meeting the different area and/or different depth wound transplantation requirements [7, 9, 59–61].Here, we list the characteristics of 3D bioprinting skins in Fig. 8.

### Challenges

The 3D bioprinting technology is emerging as a new technology for fabricating artificial skin. However, there are still significant technological challenges for the development of bio-mimetic functional skin for clinical application.

One issue faced by skin bioprinting is bioink. Quantity-seeding cells are the basic units of native skin. Although recently there have been improvements in cell culture techniques for generating cells for bioprinting; however, concerns remain whether enough cells can be readily generated for bioprinting of skin constructs for clinical applications. At present, the viability of cells can be maintained in biological materials [62], but these materials lack of bio-elasticity of native skin. A material which is suitable not only for printing 3D scaffold for seeding cells but also has the electrophysiology of native skin would be better for skin bioprinting. Therefore, optimization of materials for printing scaffolds is a major challenge for future research.

Another challenge for bioprinted skin is the lack of skin vascular network; effective vascular network is paramount to the cellular transport of oxygen and nutrients, toxic components at the same time away, so that the biological effects of skin vascular network can improve the transportation of the engineering bioskin on the wounds. Some scholars have refabricated Multi-Scale vascular networks using 3D printing technology, such as straight pipeline [63, 64] and dendritic channels [65], but these vessels still cannot address the need for blood vessels of nature skin. One reason is that natural vessels also contain cells and other components, which are the base for functional blood vessels, that is, natural vessels are different from printed blood vessels made of merely biological materials. Another reason is that human skin vascular network is so sophisticated, which requires more scholars using bioprinting technology to make breakthroughs in the field of micro vessels. Recently, Wenjie Zhang et al. [66] demonstrated that 3D-printed Scaffolds with synergistic effect of hollow-pipe structure and bioactive ions could enhance vascularized bone regeneration. Mirabella T et al. [67] introduced an approach whereby implantation of 3D-printed grafts containing endothelial-cell-lined lumens induces spontaneous, geometrically guided generation of collateral circulation in ischemic settings, and demonstrated that the vascular patches rescue perfusion of distal tissues, preventing capillary loss, muscle atrophy and loss of function. These show that 3D bioprinting technology has the potential application in bioprinting skin constructs, even though the authors have not found one study that printed blood vessels were directly applied in skin repairing.

Last but not least, the current bioprinted-skin lacks hair follicles, sweat glands, sebaceous glands, and other skin appendages, which is also the bottleneck for 3D bioprinting skin. Stem cells biological printing [56–58, 68]may be a solution to this problem; however, stem cells, epidermal stem cells, and other biological skin prints based more closely related with the skin hair follicles, still need profound works in the future.

### Outlook

Skin constructs can be fabricated using cells, collagens, or hydrogels [69] by extrusion bio-printer and LaBP. However, inject and DLP bioprinting have higher printing speed, higher cell viability (Table 2). Especially, DLP bioprinting has the properties of highest printing speed for refabricating complex structures, which has the potential of addressing the urgent need of skin constructs for grafting in clinical [15, 16, 70–72]. Future research should focus on generating skin constructs using inject and DLP-based innovative bioprinting technologies. 3D skin constructs printed from a mixture of cells, collagens, and hydrogels provides structures with limited function. How to improve the construction and function of these structures also remains a challenge at present? In addition, rigorous testing of printed skin constructs is warranted in animal models of wound healing to evaluate them for promoting wound healing but also their effect on scar formation. The knowledge gained on the therapeutic efficacy of skin constructs in animal studies would improve outcomes for the use of bioprinted skin constructs for promoting wound healing and prevention of excessive scar tissue formation in patients. Furthermore, patients who have extensive burns and full-thickness skin wounds require a treatment that results in protection of the wound during the healing process and closure of the wound in as short a time as possible. Therefore, for successful application of skin substitutes in the clinic, it is essential to decrease preparation time for bioprinting skin constructs. Early application of bioprinted skin constructs could increase recovery rate and reduce hypertrophic scar tissue [73, 74].

To cure a severely burned patient, the financial cost will be hurdles on the recovery of the burns. Because these patients often need to be specialized in burn intensive care unit for quite a long period, this period of time and materials including intensive surgical wound care, intensive care, long-term rehabilitation. It was reported that the average total medical cost per burn patient in high-income countries was $88,218 ($704–$717,306, median $44024) [2, 59, 75, 76]. The skin biological printing process [77], firstly, use a punch from the patient’s skin to obtain health organization, processing the organization after primary cells (keratinocytes, fibroblasts, melanocytes etc.) after being cultured in vitro then access to a large number of available cells, cell suspension, and ECM (hydrogel collagen, etc.). The use of biological printing ink printer to print out the preliminary skin substitutes, through the air after the page culture method, was used for skin transplantation to mature skin substitute, which will technically shorten the in-hospital time and reduce the donor site of the patients. The cost of 3D bioprinting mainly includes cell culture before printing, biological printing, print culture, operating costs etc. Therefore, the cost will reduce if the bioprinting procedure is mature and less failure rate. So far, the skin bioprinting just used in research, therefore it is difficult to calculate the cost of this skill in clinic. However, specialists are expecting its usage in foreseeable future.

## Conclusions

Bioprinting is a novel fabrication paradigm to control cellular and bio-material deposition in printed constructs, with the potential to “build” the cell-cell and cell-matrix interactions. Despite integrated structure and full functionality in skin are not included in the printed skin constructs, similar skin equivalents containing the two major cells (fibroblasts, keratinocytes) in skin have been successfully printed, thus, the missing cells, factors, structures and functions may be added inch by inch. The review of research finding described in this article [14, 36, 37, 39–41, 52–55, 59–61] demonstrate that skin bioprinting is a promising approach for an effective wound repair. Patients, especially who have extensive burns and full-thickness skin wounds, may benefit from printed skin equivalents, offering them decreased healing time and less pain, or resulting in an improved cosmetic outcome [48]. Although there are still a lot of challenges for skin bioprinting, advances in manufacturing, material science, biology, and medicine will undoubtedly move bioprinting of skin forward and address the need of native skin tissues for wound repair. In summary, skin bioprinting have the potential of realizing the fully functional skin constructs.

## Abbreviations

3D: Three dimensional
ASC: Adipose-derived stem cell
ASSG: Autologous split-thickness skin graft
ECM: Extracellular matrix
ESC: embryonic stem cell
DMD: Digital-mirror device
DOPsL: Dynamic optical projection stereolithography
FBs: Fibroblasts
KCs: Keratinocytes
LaBP: Laser-assisted BioPrinter
MSC: Mesenchymal stem cell
UV: Ultraviolet rays
